# Predicting the Risk of Total Hip Replacement by Using A Deep Learning Algorithm on Plain Pelvic Radiographs: Diagnostic Study

**DOI:** 10.2196/42788

**Published:** 2023-10-20

**Authors:** Chih-Chi Chen, Cheng-Ta Wu, Carl P C Chen, Chia-Ying Chung, Shann-Ching Chen, Mel S Lee, Chi-Tung Cheng, Chien-Hung Liao

**Affiliations:** 1 Department of Physical Medicine and Rehabilitation Chang Gung Memorial Hospital Taoyuan Taiwan; 2 Department of Orthopaedic Surgery Chang Gung Memorial Hospital Kaohsiung Taiwan; 3 Compal Electronics Smart Device Business Group Taipei Taiwan; 4 Department of Orthopaedic Surgery Pao-Chien Hospital Pingtung Taiwan; 5 Department of Trauma and Emergency Surgery Chang Gung Memorial Hospital Taoyuan City, Taoyuan Taiwan

**Keywords:** osteoarthritis, orthopedic procedure, artificial intelligence, AI, deep learning, machine learning, orthopedic, pelvic, radiograph, predict, hip replacement, surgery, convolutional neural network, CNN, algorithm, surgical, medical image, medical imaging

## Abstract

**Background:**

Total hip replacement (THR) is considered the gold standard of treatment for refractory degenerative hip disorders. Identifying patients who should receive THR in the short term is important. Some conservative treatments, such as intra-articular injection administered a few months before THR, may result in higher odds of arthroplasty infection. Delayed THR after functional deterioration may result in poorer outcomes and longer waiting times for those who have been flagged as needing THR. Deep learning (DL) in medical imaging applications has recently obtained significant breakthroughs. However, the use of DL in practical wayfinding, such as short-term THR prediction, is still lacking.

**Objective:**

In this study, we will propose a DL-based assistant system for patients with pelvic radiographs to identify the need for THR within 3 months.

**Methods:**

We developed a convolutional neural network–based DL algorithm to analyze pelvic radiographs, predict the hip region of interest (ROI), and determine whether or not THR is required. The data set was collected from August 2008 to December 2017. The images included 3013 surgical hip ROIs that had undergone THR and 1630 nonsurgical hip ROIs. The images were split, using split-sample validation, into training (n=3903, 80%), validation (n=476, 10%), and testing (n=475, 10%) sets to evaluate the algorithm performance.

**Results:**

The algorithm, called SurgHipNet, yielded an area under the receiver operating characteristic curve of 0.994 (95% CI 0.990-0.998). The accuracy, sensitivity, specificity, and *F*_1_-score of the model were 0.977, 0.920, 0932, and 0.944, respectively.

**Conclusions:**

The proposed approach has demonstrated that SurgHipNet shows the ability and potential to provide efficient support in clinical decision-making; it can assist physicians in promptly determining the optimal timing for THR.

## Introduction

Deteriorating hip joint disorder causes disability worldwide, and hip osteoarthritis alone ranks as the 11th highest contributor [1-3]. Total hip replacement (THR) [4] is considered the international gold standard for treating refractory degenerative and rheumatologic hip disorders [5]. The indication criteria for THR in guidelines still appear unclear, mentioning domains like pain, function, radiologic change, and the limited effectiveness of nonoperative therapy, but they lack specific cut-off values or ranges [6,7]. THR has been considered one of the most cost-effective and successful orthopedic interventions currently available [8,9], which can improve pain and function in patients for whom it is indicated [10]. More than 1 million THRs are performed each year [11]. Identifying patients who are more likely to receive THR in the short term is of interest, as pain and self-reported functional status in patients might not deteriorate with a shorter wait time for surgery [12].

The timing of the decision to refer patients for surgical consultation is vital for primary health providers. Better preoperative hip function in patients is indicative of better postoperative functional outcomes [13], and a delayed operation could result in poorer outcomes [14]. The decision and timing of referring a patient to a hip specialist are important for the general practitioner. Referring patients too early is not recommended for those patients who respond well to conservative treatment, as it could result in longer waiting times for the patients who truly require THR referrals. A late referral might also delay the timing of the operation, affecting the surgical outcome and patient prognosis, and it could decrease patients’ quality of life [15,16]. Additionally, conservative treatments like intra-articular corticosteroid injection may result in higher odds of prosthetic joint infection for patients who are scheduled to receive THR within 3 months or even longer [17,18]. Therefore, identifying patients who should receive operations in the short term is crucial for primary health providers [19].

Anteroposterior pelvic radiography is the most commonly used primary diagnostic tool for assessing hip joint conditions, such as fractures, osteonecrosis, and degenerative disorders [20,21]. These assessments provide critical information to guide and offer a prognosis as well as treatment options [22]. A recent systematic review [23] suggested that information obtained from PXRs, including higher Kellgren and Lawrence grades, superolateral femoral head migration, and subchondral sclerosis, were predictive of a faster progression to THR [23]. However, accurate recognition of these features needs expertise and is time-consuming. An automated detection model for predicting THR within a short-term period has potential benefits, including increasing efficiency, reducing delayed referrals, and improving patient outcomes.

Significant breakthroughs in medical imaging applications have been achieved in the field of deep learning (DL), improving the speed of diagnosis and decision-making with comparable efficacy to that of professional clinicians [24-28]. DL has been used to detect lesions in pathologic images, electrocardiography, and retinography [29-32]. In the field of hip studies, DL techniques have been applied to detecting hip fractures [33], diagnosing osteoarthritis [34], grading the severity of osteoarthritis [35], and predicting patient-specific payment models [36,37].

Artificial intelligence (AI) has been suggested not only for aiding in diagnosis prediction but also for practical wayfinding in clinical decision-making [38]. Although the advancement of medical AI in the orthopedic field is on the rise, DL applications in practical wayfinding, such as short-term THR prediction, are still lacking. In this study, we use a DL-based framework to develop SurgHipNet, a fully automated diagnosis assistance system, using PXRs to obtain THR prediction within 3 months. The goal of this study was to develop and validate a DL model that automatically extracts radiographic features of the hips and assists primary health providers in identifying patients who need THR in the short term for early referral.

## Methods

### Data Acquisition

We extracted the PXRs and surgical reports of all patients who underwent THR in Linkou Chang Gung Memorial Hospital (CGMH) from August 2008 to December 2017. This data collection consisted of 3013 PXRs within 3 months before THR surgery (THR group). To identify the non-THR group, we used the trauma data bank in CGMH to search for patients who had PXRs during the same time period as the THR group. From these records, we identified 1630 individuals who did not receive any hip surgery at our hospital within the following 3 months, as confirmed by their medical records. These individuals were included in our study as the non-THR group. We excluded images depicting previous hip surgery with foreign bodies, hip fractures, and pediatric skeletal images, as well as those with poor image quality.

### Ethical Considerations

For deidentification purposes, each image file name was converted to a serial number, and the upper and lower sections of the images containing patient privacy information, such as names, medical record numbers, and date of birth, were cropped out. For the fully anonymized reports and radiographs used in this study, the requirement to obtain informed consent was waived. The Internal Review Board of CGMH approved this study (201801784B0).

### Data Annotation

**Figure 1:**
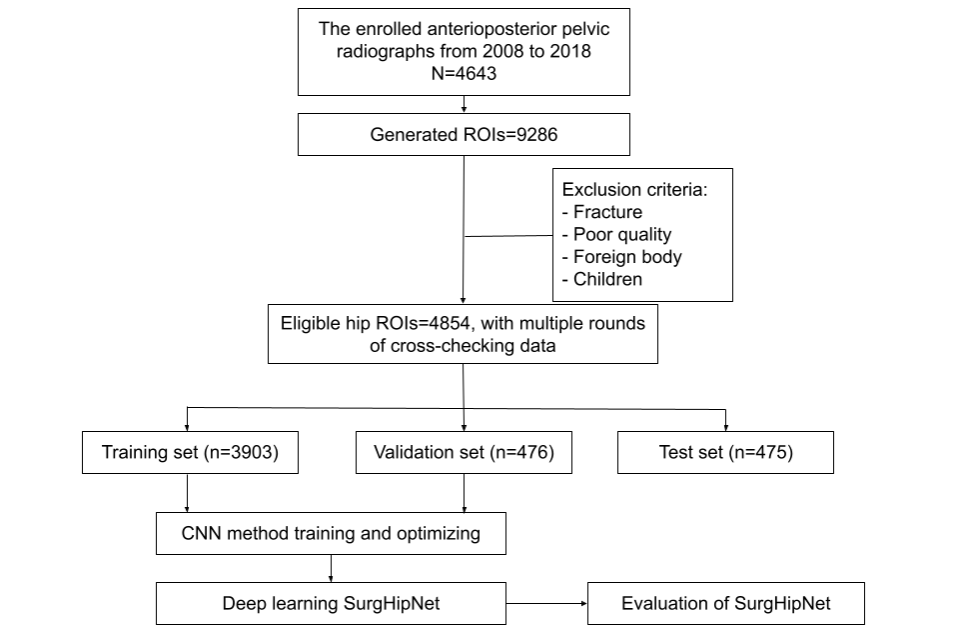
Flowchart showing participant selection from the database. CNN: convolutional neural network; ROI: region of interest.

The final data set consisted of 4854 hip joints, using 4643 weight-bearing anterior-posterior PXRs from 4643 participants (Figure 1). Hip regions of interest (ROI) were split into training (n=3903, 80%), validation (n=476, 10%), and independent test (n=475, 10%) data sets. We used the training data set for model training and performed 20-fold cross-validation. Then we used the validation set to adjust the hyperparameters to elevate the performance and prevent overfitting. Lastly, we used the independent test data set (n=475) to validate the final performance.

The images were initially labeled as THR or non-THR according to the surgical reports. For the THR group, we reviewed all surgical reports to ensure that patients had undergone THR and then acquired the corresponding images for training. For the non-THR group, we could only confirm that patients had not received THR within the subsequent 3 months at our hospital. For joint localization training, we developed a labelless practical framework to automatically detect hips. Bounding boxes were placed at the center of the femoral head. To annotate the hip ROI, we used 3 annotators trained to place square bounding boxes approximately centered at the femoral head or the artificial hip joint with customized graphical user interface software. All the labeled ROIs in the data set were visually reviewed by 1 physician with 15 years of clinical experience, and the ROI annotators used the same rules to annotate the data sets. The hip ROIs were cropped from PXRs by single-shot detector–based algorithms. A preprocessing procedure including cropping of square regions and mirroring of the left hip ROI (a process of flipping the left hip ROI into mirrored left hip ROIs) was needed. The square hip ROI was resized to 224 224 pixels with an 8-bit grayscale color format to reduce complexity and computation. For nonsquare input radiographs, the image was padded to achieve a square size, with zero values added to the width or height to ensure that the convolution operation preserves the aspect ratio of the hip and pelvis shape in the radiograph. The detailed framework of the development method has been described in Multimedia Appendix 1 and a previous study [39]. The hip ROIs were then inputted into a further network for classification.

### SurgHipNet Architecture, Data Preprocessing, and Model Training

The SurgHipNet was designed based on ResNet 101 and pretrained using ImageNet. It is used with PyTorch v0.4 and fastai API 2020 implementation and runs on CUDA 9.0 within the Ubuntu 16.04 operating system, powered by 1 Nvidia Tesla V100 graphic processing unit. For model training, we used Adam optimizer and focal loss, with α=.5 and γ=5. The training schedule used a cyclical learning rate and the 1-cycle policy, where 2 cycles were trained with the first 10 epochs on the last layer and 10 additional epochs on the last 2 layers. We used 1 Nvidia 1080Ti graphic processing unit on the Ubuntu 16.04 operating system.

A 20-fold cross-validation approach was used on the data set. We used the classification error rate as the loss function and Adam optimizer (β_1_=.9 and β_2_=.99) and added a final BatchNorm layer. We trained the model with minibatches of size 16 and used a cyclical learning rate strategy and 1-cycle policy, where we first trained the last layer with differential learning rates, using max_lr=0.003 for 10 cycles; we then trained all layers using the same learning rate setting for 10 cycles. The details of the comparative experiments are listed in Multimedia Appendix 2. We augmented the data during training with the following fastai augmentation settings:



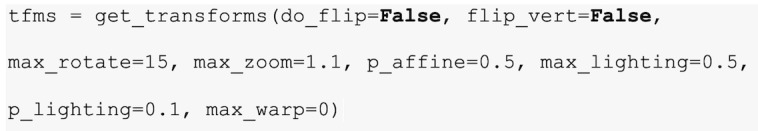



### Algorithm Prediction Visualization

For the SurgHipNet models that predict the need for THR, we also used Gradient-weighted Class Activation Mapping (Grad-CAM) to generate a heatmap activated by the model for the pathologic areas of the hip joint space or femoral head. This heatmap provided evidence that the model accurately identified the potential pathologic sites. This method ensures that the algorithm detects and classifies based on the area around the femoral head, rather than other segmentations of the image, as previous experiences have shown [40].

### SurgHipNet Evaluation and Statistical Analysis

All statistical analyses were carried out using R 3.6.3 (R Core Team) with the packages “pROC,” “tableone,” “caret,” and “ggplot2.” We reported overall accuracy, sensitivity, specificity, positive and negative predictive value, *F*_1_-score, and the area under the receiver operating characteristic curve (AUC) along with the 95% CIs, which were estimated using bootstrapping with 2000 replicates to evaluate the performance of SurgHipNet.

## Results

### Data Characteristics

In this study, we used a total of 4854 hip ROIs, including 1138 ROIs with THR and 3716 ROIs without THR, to develop our algorithm. The characteristics and epidemiology of the images are presented in Table 1. We included all the ROIs and split the data set into training (n=3903, 80%), validation (n=476, 10%), and testing sets (n=475, 10%).

**Table 1:** Characteristics of etiology and grading of the data sets.

Group and data set etiology and grades			Training data set (n=3903), n (%)		Validation data set (n=476), n (%)		Testing data set (n=475), n (%)
**THR^a^ group (n=1383)**							
	Avascular necrosis-1	16 (0.4)		3 (0.6)		4 (0.8)	
	Avascular necrosis-2	189 (4.8)		17 (3.6)		23 (4.8)	
	Avascular necrosis-3	232 (5.9)		36 (7.6)		26 (5.4)	
	Avascular necrosis-4	282 (7.2)		28 (5.9)		32 (6.7)	
	Avascular necrosis-5	2 (0.05)		N/A^b^		N/A	
	Osteoarthritis-1	8 (0.2)		7 (1.5)		1 (0.2)	
	Osteoarthritis-2	23 (0.6)		7 (1.5)		4 (0.8)	
	Osteoarthritis-3	70 (1.8)		6 (1.3)		4 (0.8)	
	Osteoarthritis-4	82 (2.1)		2 (0.4)		10 (2.1)	
	Osteoarthritis-5	3 (0.08)		1 (0.2)		1 (0.2)	
	Other etiologies	17 (0.4)		1 (0.2)		1 (0.2)	
**Non-THR group (n=3716)**							
	Normal	2246 (57.5)		283 (59.4)		269 (56.6)	
	Osteoarthritis-1	733 (18.8)		85 (17.9)		100 (21.1)	

^a^THR: total hip replacement.

^b^N/A: not applicable.

**Figure 2:**
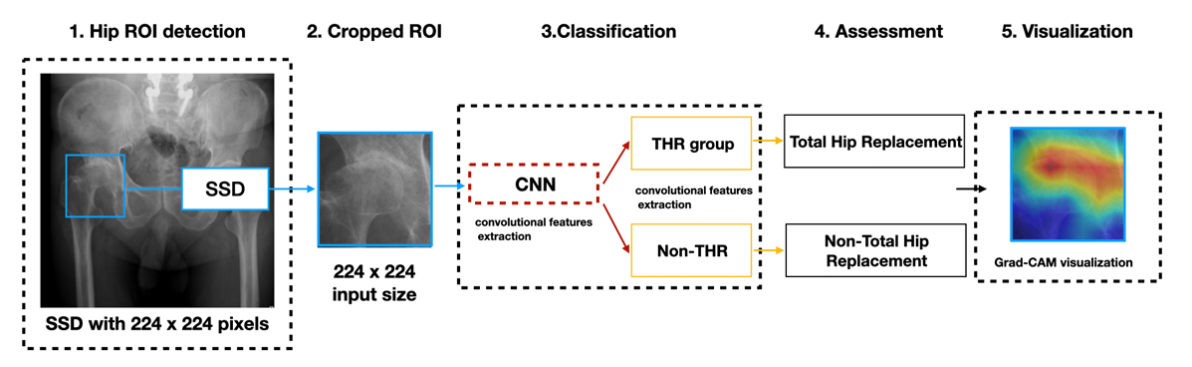
The SurgHipNet pipeline. The input images detected the hip region of interest (ROI) first, and then, we cropped the hip area as our target ROI. Then the cropped images were inputted into a convolutional neural network (CNN) to be further classified into total hip replacement (THR) or non-THR groups. Finally, a Gradient-weighted Class Activation Mapping (Grad-CAM) was applied for the final visualization. SSD: single-shot detector.

In the beginning, each hip region in a radiograph was labeled by an autonomic hip detection system [39]. The cropped ROI was used with a convolutional neural network–based algorithm for classification into the THR or non-THR groups. Furthermore, the Grad-CAM technique was adapted for visualization. The deep neural network learning architecture is presented in Figure 2.

### SurgHipNet Performance on the Independent Test Data Set

**Figure 3:**
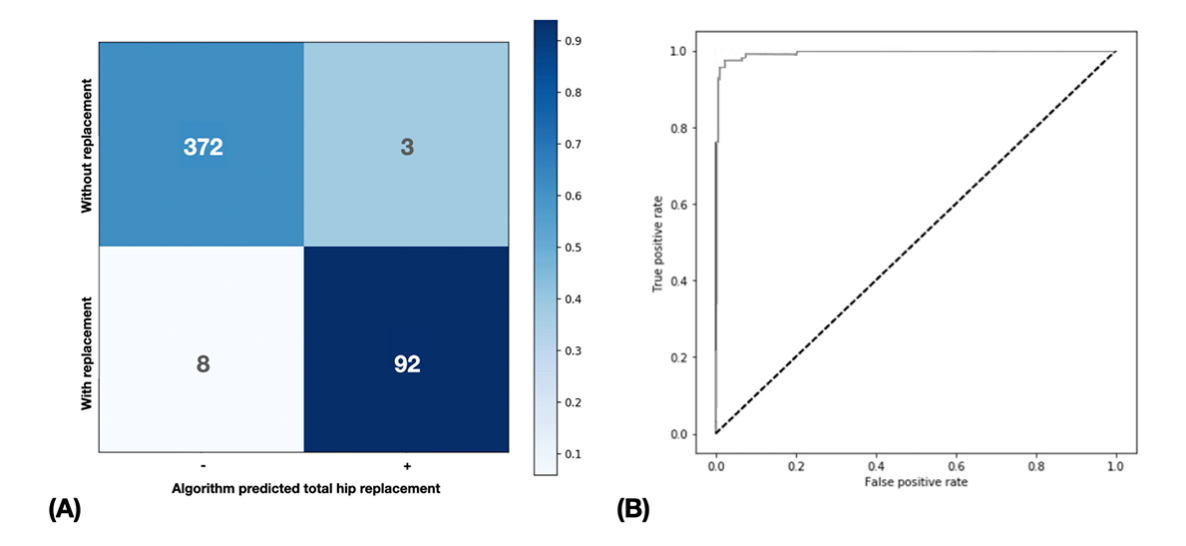
(A) The confusion matrix plot of the SurgHipNet algorithm. (B) The receiver operator characteristic curve and the summary of classification results on other independent test data sets comprising 475 ROIs. The area under the receiver operator characteristic curve was 0.9947.

We used the isolated testing data set to evaluate the performance of SurgHipNet. It predicted the THR with an acceptable performance; the overall accuracy, sensitivity, specificity, and AUC were 0.977, 0.9200, 0.992, and 0.994 (95% CI 0.990-0.998), respectively (Table 2; Figure 3).

**Table 2:** The performance of SurgHipNet classification results in the testing data set.

Features	Values
True positive	92
True negative	372
False positive	3
False negative	8
Accuracy	0.977
Prevalence	0.211
Sensitivity	0.920
Specificity	0.992
Positive predictive value	0.968
Negative predictive value	0.979
*F*_1_-score	0.944
Area under the receiver operating characteristic curve (95% CI)	0.994 (0.990-0.998)

### Model Interpretation Using Grad-CAM Visualization

**Figure 4:**
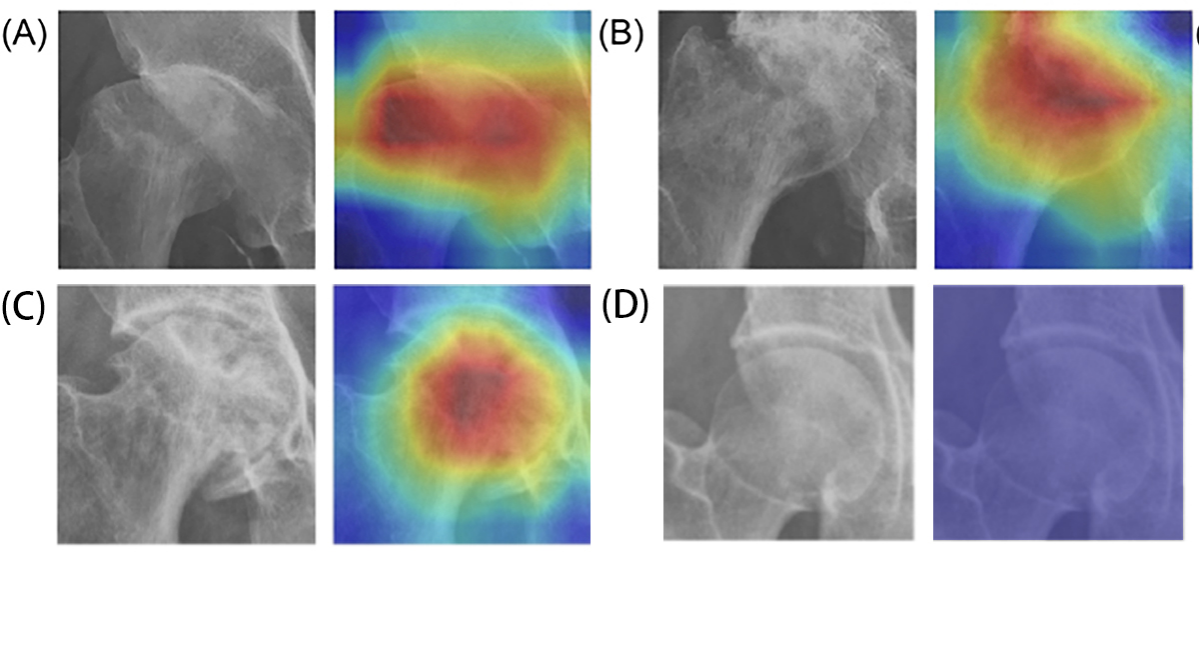
Representative Gradient-weighted Class Activation Mapping (Grad-CAM) visualization examples (the total hip replacement group). (A) The hip region of interest shows the collapse of the femoral head and the narrowing of the joint space. (B) The heatmap highlights the collapsed femoral head with the destruction of the hip space. (C) The heatmap highlights the destruction of the femoral head. (D) Normal hip region of interest visualization.

Grad-CAM was applied after the last convolutional layer of the model and then overlaid with the radiograph to show the relevance of specific areas for the model’s classification process. Figure 4 represents 3 ROIs through visualizations, each of which was classified using the THR prediction model. The heatmap identified either a narrowing of the hip joint space or the presence of a destructive femoral head or acetabular rims. The last image in Figure 4 is the ROI of the hip, classified with a normal prediction.

**Figure 5:**
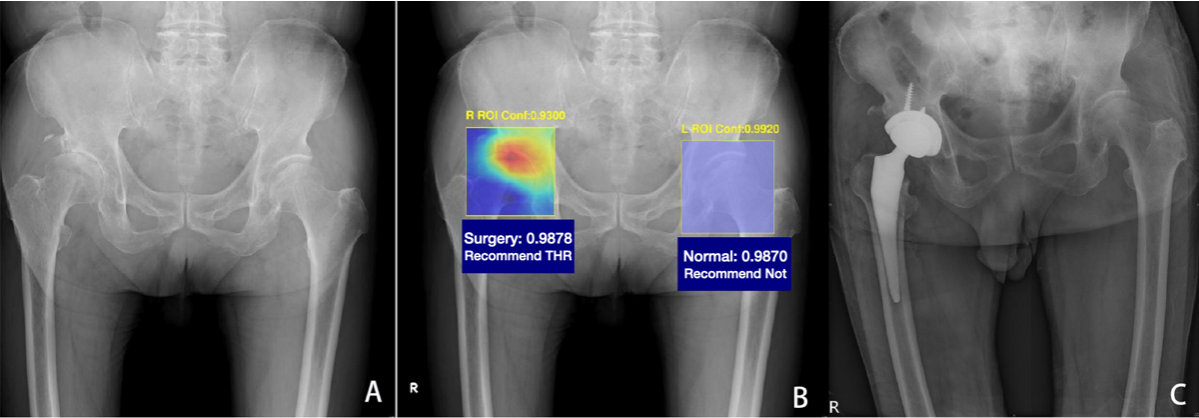
The demonstration of SurgHipNet (the total hip replacement group). (A) The input image of plain pelvic film of a patient with the right hip narrowing of the joint space and femoral head collapse. (B) SurgHipNet detects the hip region of interest and presents the predictive value for a hip replacement recommendation. The right hip joint was predicted to need surgery, and the left one was predicted to be a normal hip joint. The heatmap showed the right femoral head collapse with the joint space narrowing. (C) 3 months later, the post-THR plain film of this patient. Conf: configuration; L: left; R: right. ROI: region of interest.

After all the processing steps, the ROIs were integrated into the previous pelvic film to create a predictive image as shown in Figure 5 for clinical physicians to review.

## Discussion

### Principal Findings

In this study, it has been demonstrated that the SurgHipNet system can assist frontline doctors in identifying patients who have a high possibility of needing to receive THR within 3 months. In the testing set, the accuracy, sensitivity, specificity, and AUC for the prediction of THR were 0.977, 0.920, 0.992, and 0.994, respectively. To mitigate the “black box” effect, we applied Grad-CAM to generate a heatmap that shows the affected areas of the joint, further drawing the user's attention to potential lesions detected by SurgHipNet. To the best of our knowledge, this is the first study that provides THR prediction. Frontline physicians can timely refer selected patients to orthopedic experts by recommendations from the SurgHipNet model. It has the potential to be used in a wide range of medical imaging applications.

Musculoskeletal disorders, including osteoarthritis, are the most significant contributors to disability worldwide. Hip osteoarthritis is one of the most commonly affected large joints, and its global prevalence continues to rise due to factors such as aging and obesity [3,16]. Conservative treatments, including pharmacologic treatment, exercise, physical therapy, weight reduction, proper footwear, and assistive devices, are the primary therapeutic options [41-43]. It is still unclear who should be referred to THR. Guidelines only suggest that THR is suitable for patients with intractable pain, failed nonsurgical treatments, and functional limitations, and patients often receive the operation with delay [43,44]. However, more established evidence suggests that early surgical intervention reduces associated complications and improves the quality of life for patients experiencing hip discomfort [45]. Higher postoperative levels of range of motion and muscle strength were also observed in patients who had THR surgery earlier, compared to patients who had the surgery later in their disease progression [46]. THR has proven to be cost-effective in the management of hip osteoarthritis, especially for those aged 30-80 years [16].

Medical AI is changing the health care system, and DLs have been used to detect lesions in pathologic images, electrocardiography, and radiography [29]. These algorithms have demonstrated remarkable achievements in disease detection and prediction, achieving a similar level of accuracy to that of experienced physicians. AI has also developed rapidly in the orthopedic field, with applications in image diagnostics, prediction of surgical risk, clinical decision-making, and outcome prediction [27,33,47]. DL has shown its potential in investigating hip arthropathy. Xue et al [34] used a VCG-16 model for hip osteoarthritis classification. Their model showed high sensitivity, specificity, and accuracy compared to chief physicians with 10 years of experience. von Schacky et al [35] recently developed a multitasking DL model for the grading of hip osteoarthritis severity, which also showed a comparable performance with that of expert radiologists. Zhang et al [48] developed a DL model for the diagnosis of developmental dysplasia of the hip, which was highly consistent and more effective in comparison with clinician-led diagnoses.

Recently, medical AI has been suggested to be not only a diagnosis prediction but also a wayfinding tool in clinical decision-making [38]. Leung et al [49] developed a DL prediction model for the prediction of total knee replacement for osteoarthritis progression evaluation. Our study used DL to predict the timing of THR in months, providing practical clinical utility. To our knowledge, this is the first proposed DL model that predicts THR. With this developed model, frontline physicians can effectively refer selected patients to experts in hip replacement, which is a wayfinding support in clinical decision-making.

### Study Limitations

The proposed algorithm’s use can improve diagnostic accuracy for patients who may need THR, and it can support decision-making in surgical consultations. However, there are still some limitations. First, the algorithm currently does not integrate clinical information, which differs from clinical practice considerations. Decisions regarding arthroplasty might be affected by clinical presentations, such as rest pain, range of movement, underlying conditions [50], as well as the economic and environmental conditions of the patient [51]. In these situations, patients would not necessarily undergo surgery even if surgery is recommended by SurgHipNet’s review of the PXRs. On the other hand, there are occasions where SurgHipNet will advise against performing THR, but the patient will still need surgical intervention; for example, if they need to partake in more activity or if their pain and restricted movement prevent them from being able to access an ambulance. As we suggested, the goal of this developed algorithm is to assist the decision-making process for referring patients to THR surgeons without delay, but it is not intended to be a substitution for a clinician’s judgment [52]. Using their expertise, clinicians will make a final suggestion after integrating image findings, the patient’s clinical condition, the patient’s willingness, and the algorithm results. Future studies could attempt to integrate patients' clinical data into the system, thereby enhancing the algorithm’s comprehensiveness. The second limitation is that the limited number of physicians participating in this evaluation could result in an underpowered study. Third, the indication for performing a PXR among individuals in the non-THR group was not clear, which could potentially introduce confounding results. Fourth, because the algorithm focused on proposing the need for THR rather than detecting the severity of patients’ conditions, the training data, labeling, and design were limited to using DL to determine the severity of the degenerative joint disease. Unlike previous studies [35,53] that focused on providing high-resolution heatmaps, further processing is not recommended. Through our study, the physician's attention can be directed toward the ROI within the hip joint; this ensures that the algorithm makes assessments and predictions based on the features of the hip lesion site. However, the resolution might be relatively coarse, leading to possible false negative predictions (Multimedia Appendix 3). In the future, adding labels around the joints to create better heatmaps could increase the confidence of physicians using this model. Further, any hip surgeries that patients in the non-THR group might have undergone in other hospitals, which cannot be detected through our system’s medical records, were also likely to potentially contribute to confounding results. Finally, the independent test data set images were only from one institution; even though we already performed validation, the overfitting could not be prevented. A prospective multicenter study should be completed to investigate the system’s function in the real world.

### Conclusions

In conclusion, we developed SurgHipNet to predict the probability of THR within 3 months. This is the first algorithm designed to suggest THR referrals. Future prospective studies are required to validate whether applying SurgHipNet as a computer-aided tool in a clinical environment leads to more precise diagnoses and facilitates patient management.

## Appendix

Multimedia Appendix 1 The hip region of interest detection algorithm description.

Multimedia Appendix 2 The comparison experiments for the SurgHipNet development.

Multimedia Appendix 3 An example of incorrect classification of SurgHipNet.

## Abbreviations

AI: artificial intelligence
AUC: area under the receiver operating characteristic curve
CGMH: Chang Gung Memorial Hospital
DL: deep learning
Grad-CAM: Gradient-weighted Class Activation Mapping
PXR: pelvic radiograph
ROI: region of interest
THR: total hip replacement
