# Supplementary material for: Predicting the Risk of Total Hip Replacement by Using A Deep Learning Algorithm on Plain Pelvic Radiographs: Diagnostic Study
Source: JMIR Form Res. 2023 Oct 20;7:e42788. doi: 10.2196/42788 (PMC10625092; doi:10.2196/42788)
Supplement: Multimedia Appendix 1 [file formative_v7i1e42788_app1.docx]

**Appendix 1 : The Hip ROI detect algorithm description.**

Section 1: HipRD Data Acquisition

In addition to Chang Gang Osteoarthritis (CGOA) dataset with high resolution radiographs, we the second Osteoarthritis Initiative Hip (OAIH) dataset containing with additional 3,008 low to medium resolution radiographs from Osteoarthritis Initiative for training and validation of the hip region detection module. The OAIH dataset is a consolidated pelvic radiograph dataset extracted from subset of data from the Osteoarthritis Initiative (OAI), a prospective, longitudinal, and observational study of knee osteoarthritis [OAI]. The OAI project recruited 4,796 participants from February 2004 to May 2006 to form a baseline cohort (58% female and ranged in age from 45-79 at time of recruitment) and have retained most patients for follow up visits in clinic with either biospecimen collection and/or imaging at full-limb, hand, knee, or pelvis at 12-month, 24-month, 36-month, and 48-month, 72-month, and 96-month. In total we identified 11354 AP pelvic radiographs (4707 at baseline; 64 at 12-month visit.

The third GIS dataset was acquired through Google image search engine.  After manual curation to filter out irrelevant or poor-quality images. We collected 101 radiographs with a wide size distribution ranging from 225 x 225 pixels to 4256 x 3495 pixels as independent test data for the study.

Section 2: HipRD Network Architecture, Data Preprocessing and Model Training

We utilized PyTorch v.031 and fastai API 2018 implementation and CUDA 9.0 on Ubuntu 16.04 operating system with one Nvidia 1080Ti GPU. We utilized single-shot multibox detector (SSD) (refer [SSD]) with ResNet-101 (refer [ResNet]) backbone pre-trained on ImageNet (refer [ImageNet]) and resigned of the head structure by a customized anchor layout. The specially designed anchors considered the object sizes relative to the whole image in the datasets and ensures high performance of hip region detection.

For non-square input radiograph, the image is padded to square size with zero values either on the short side of width or height to ensure that the convolution operation preserves the aspect ratio of the hip and pelvis shape in the radiograph. The square image is then resized to 224 x 224 pixels with an 8-bit grayscale color to reduce the complexity and computation.

We randomly split the combined CGOA and OAIH dataset into 90% for training and 10% for validation and used all 101 radiographs in the GIS dataset as the independent test dataset. We used focal loss with (α=0.25, γ=5) (refer [Focal]) and Adam optimizer with (β1=0.9, β2=0.99) (refer [Adam]). We trained the model with minibatches of size 16 and utilized cyclical learning rate and one cycle policy (refer [Cycle]), where we first trained the last layer with learning rate lr=0.01, learning array lrs [lr/100,lr/10,lr] with 40 cycles, then trained the last two layers with a smaller learning rate lr=0.0025 with 40 cycles, and followed with unfreezing all layers with smaller learning rate lr=0.0025 with 40 cycles. We augmented the data during training by applying random rotations of up to 3 degrees with 0.9 probability and random lighting with (balance=0.5, contrast=0.5).

Section 3: HipRD Evaluation and Statistical Analysis

We used the standard metric, Intersection over Union (IoU) for comparing the predicted bounding box Bpred and ground truth bounding box Bgt

| IoU$= \frac{B_{pred}\cap B_{gt}}{B_{pred}\cup B_{gt}}$ |  |
| --- | --- |

where B_pred∩B_gt denotes the intersection of the predicted and ground truth bounding boxes and B_pred∪B_gt denotes their union. In addition, we reported the associated confidence calculated by the SSD algorithm for each predicted bounding box, average IoU, average confidence, minimal confidence, and AP50 (average precision with IoU > 0.5), where the threshold of 50% was set for poor bounding boxes detection which may cause issues for downstream analysis.

Section 4: HipRD computational performance

We first evaluated the task of hip region detection and investigated if DNN-based approach can identify the hip ROIs correctly. The detailed performance was presented in previous study. [1]

Reference :

1 . [Liu F-Y, Chen C-C, Cheng C-T, Wu C-T, Hsu C-P, Fu C-Y, et al. Automatic Hip Detection in Anteroposterior Pelvic Radiographs—A Labelless Practical Framework. Journal of Personalized Medicine. 2021 Jun 7;11(6):522.](http://paperpile.com/b/EQY1aU/TJzK)
