# Supplementary material for: Predicting the Risk of Total Hip Replacement by Using A Deep Learning Algorithm on Plain Pelvic Radiographs: Diagnostic Study
Source: JMIR Form Res. 2023 Oct 20;7:e42788. doi: 10.2196/42788 (PMC10625092; doi:10.2196/42788)
Supplement: Multimedia Appendix 2 [file formative_v7i1e42788_app2.docx]

**Appendix 2. The comparison experiments for SurgHipNet Development.**

| Exp index | CNN | Hip ROI detection method | Total ROI number | Number of Training ROIs | Number of fold cross validation | optimizer | accuracy |
| --- | --- | --- | --- | --- | --- | --- | --- |
| exp001 | ResNet 34 | manual | 3903 | 1950 | 2 | SGD | 0.9844 |
| exp002 | ResNet 34 | manual | 3903 | 3122 | 5 | SGD | 0.9877 |
| exp003 | ResNet 34 | manual | 3903 | 3512 | 10 | SGD | 0.9885 |
| exp004 | ResNet 34 | manual | 3903 | 3707 | 20 | SGD | 0.9905 |
| exp005 | ResNet 101 | manual | 3903 | 3864 | 100 | SGD | 0.9900 |
| exp006 | ResNet 101 | manual | 3903 | 1950 | 2 | SGD | 0.9877 |
| exp007 | ResNet 101 | manual | 3903 | 3122 | 5 | SGD | 0.9908 |
| exp008 | ResNet 101 | manual | 3903 | 3512 | 10 | SGD | 0.9915 |
| exp009 | ResNet 101 | manual | 3903 | 3707 | 20 | SGD | 0.9923 |
| exp010 | ResNet 101 | manual | 3903 | 3707 | 20 | Adam | 0.9928 |
| exp011 | ResNet 101 | adam crop 01 | 3903 | 3707 | 20 | Adam | 0.9869 |
| exp012 | ResNet 101 | adam crop 02 | 3903 | 3707 | 20 | Adam | 0.9918 |
| exp013 | ResNet 101 | adam crop 03 | 3903 | 3707 | 20 | Adam | 0.9926 |

CNN: Convolutional neural network; ROI : Region of interest; SGD: Stochastic gradient descent.
