# Supplementary material for: Predicting the Risk of Total Hip Replacement by Using A Deep Learning Algorithm on Plain Pelvic Radiographs: Diagnostic Study
Source: JMIR Form Res. 2023 Oct 20;7:e42788. doi: 10.2196/42788 (PMC10625092; doi:10.2196/42788)
Supplement: Multimedia Appendix 3 [file formative_v7i1e42788_app3.docx]

**Appendix 3. The Example of Incorrect classification of SurgHipNet**

**S3a : SurgHipNet predicts no need for surgery on both hips. However, this patient underwent right total hip replacement within three months.**

**
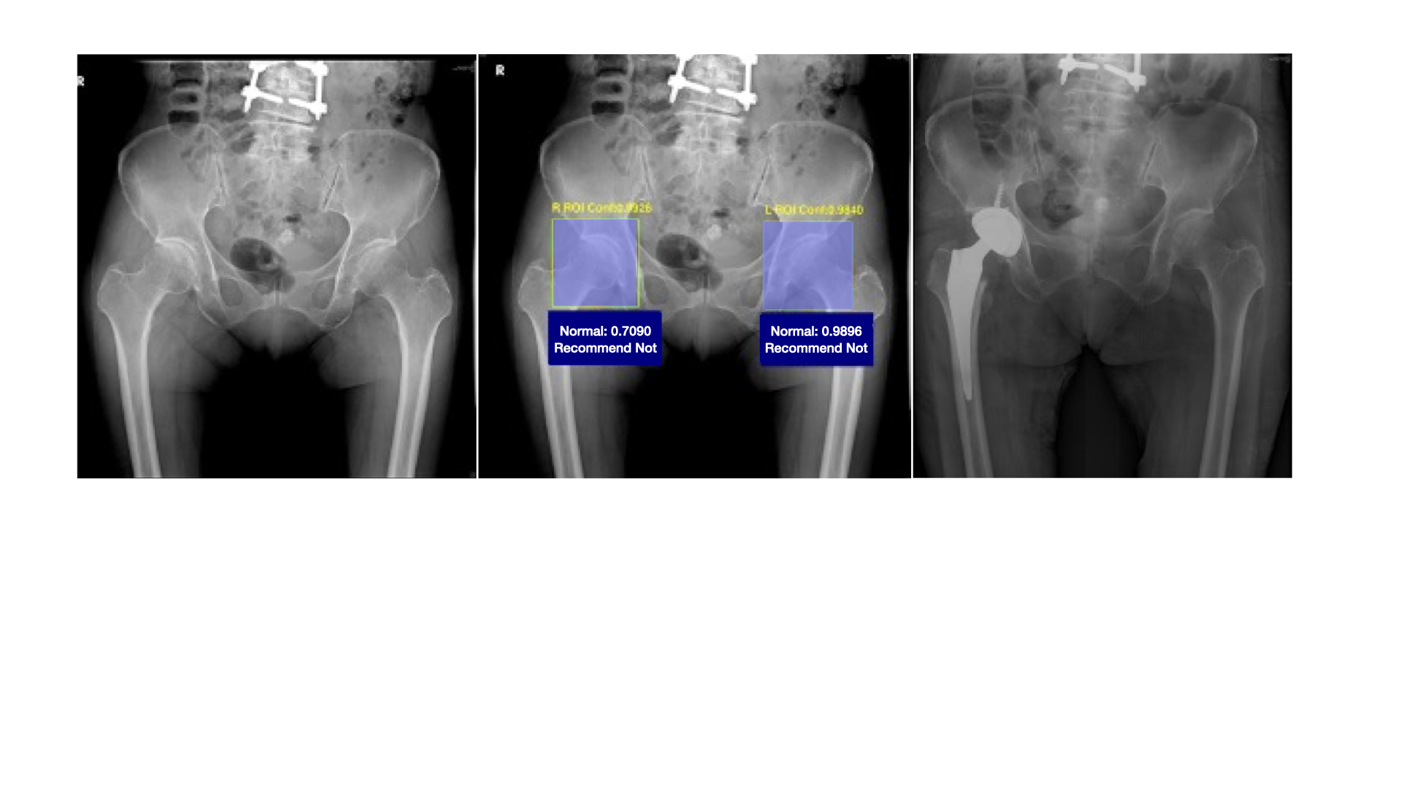
**

**S3b :** **The patient suffered a fracture of the right femoral neck, but SurgHipNet could not classify this patient into the THR group because the fracture features were not recognized well in this case.**

**
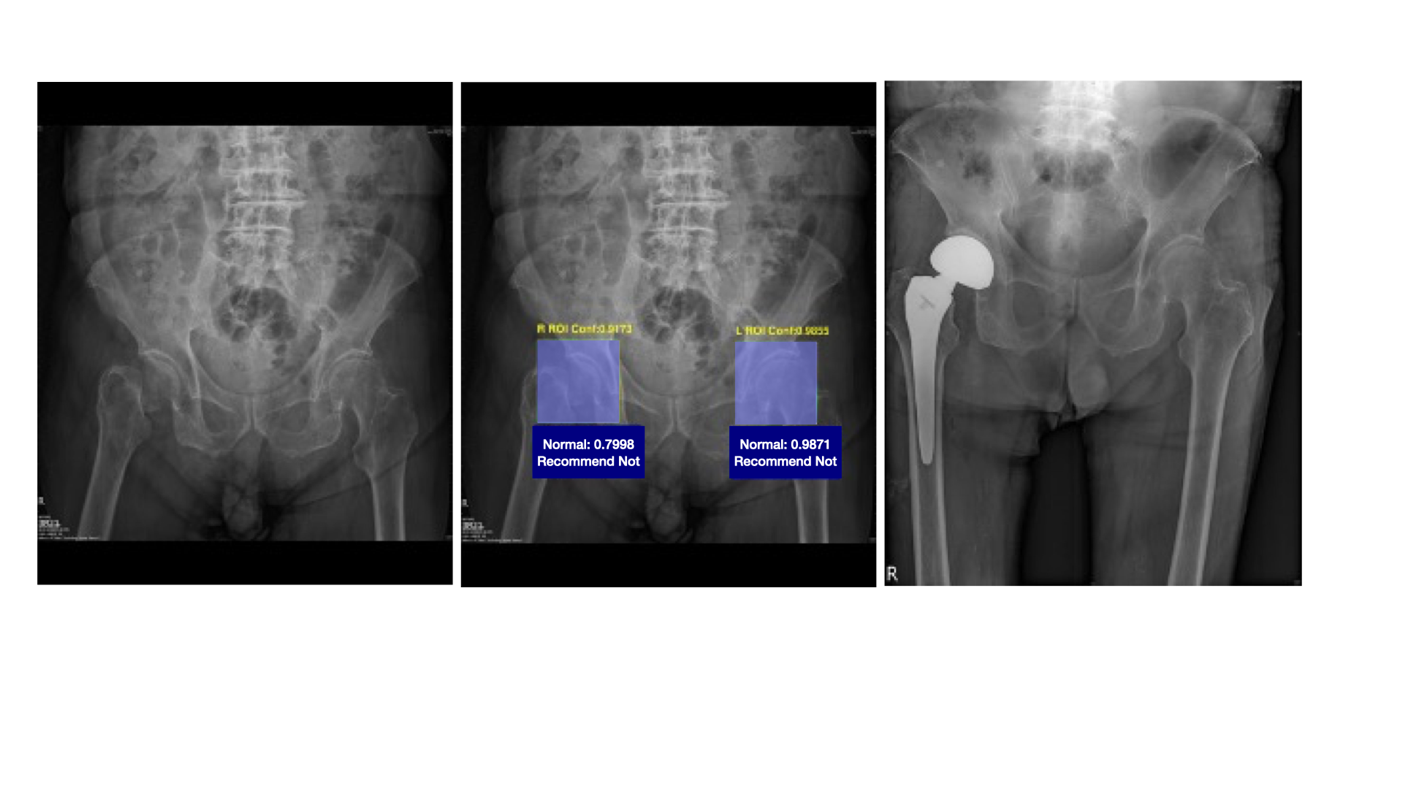
**
